# The Prognostic Value of the Albumin to Gamma-Glutamyltransferase Ratio in Patients with Hepatocellular Carcinoma Undergoing Radiofrequency Ablation

**DOI:** 10.1155/2021/3514827

**Published:** 2021-11-19

**Authors:** Wenfeng Liu, Feng Zhang, Bing Quan, Miao Li, Shenxin Lu, Jinghuan Li, Rongxin Chen, Xin Yin

**Affiliations:** ^1^Liver Cancer Institute, Zhongshan Hospital, Fudan University, Shanghai 200032, China; ^2^National Clinical Research Center for Interventional Medicine, Shanghai 200032, China

## Abstract

Albumin to gamma-glutamyltransferase ratio (AGR) is a newly developed biomarker for the prediction of patients' prognosis in solid tumors. The purpose of the study was to establish a novel AGR-based nomogram to predict tumor prognosis in patients with early-stage HCC undergoing radiofrequency ablation (RFA). 394 hepatocellular carcinoma (HCC) patients who had received RFA as initial treatment were classified into the training cohort and validation cohort. Independent prognostic factors were identified by univariate and multivariate analyses. The value of AGR was evaluated by the concordance index (*C*-index), receiver operating characteristic (ROC) curves, and likelihood ratio tests (LAT). Logistic regression and nomogram were performed to establish the pretreatment scoring model based on the clinical variables. As a result, AGR = 0.63 was identified as the best cutoff value to predict overall survival (OS) in the training cohort. According to the results of multivariate analysis, AGR was an independent indicator for OS and recurrence-free survival (RFS). In both training cohort and validation cohort, the high-AGR group showed better RFS and OS than the low-AGR group. What is more, the *C*-index, area under the ROC curves, and LAT *χ*^2^ values suggested that AGR outperformed the Child-Pugh (CP) grade and albumin-bilirubin (ALBI) grade in terms of predicting OS. The AGR, AKP, and tumor size were used to establish the OS nomogram. Besides, the results of Hosmer-Lemeshow test and calibration curve analysis displayed that both nomograms in the training and validation cohorts performed well in terms of calibration. Therefore, the AGR-based nomogram can predict the postoperative prognosis of early HCC patients undergoing RFA.

## 1. Introduction

Primary liver cancer can be divided into three types: hepatocellular carcinoma (HCC), intrahepatic cholangiocarcinoma, and combined hepatocellular and cholangiocarcinoma [1]. HCC is one of the most common malignancies in humans and the second leading cause of cancer-related deaths in men in developing countries [2, 3]. The high incidence of HCC in Asia is closely related to hepatitis B virus (HBV), with more than 5 percent of people being chronically infected [4], whereas alcoholism and hepatitis C virus (HCV) infection are more closely related to HCC in Western countries [5].

Tumor ablation is a widely accepted treatment choice for patients with early stage HCC. Ablation induces tumor necrosis through temperature changes or injection of chemical reagents such as ethanol. Among them, radiofrequency ablation (RFA) is the preferred ablation technique because it provides better disease control than percutaneous ethanol injection [6]. This difference is particularly pronounced in tumor nodules with a diameter of more than 2 cm. In HCC patients with Child-Pugh (CP) A, the survival rate after ablation is similar to those undergoing surgical resection [7]. In addition, previous studies have proved that the long-term therapeutic effect of RFA for patients with small HCC is equivalent to liver resection and liver transplantation [8, 9].

The prognostic prediction of tumors is particularly important and meaningful. Tumor-Node-Metastasis (TNM) system and CP classification are widely used to assess the clinical prognosis of HCC patients. However, it has been recognized that there are differences in the prognosis of patients with the same TNM stage and CP grade [10]. Other pathological features and tumor markers such as tumor differentiation, tumor size, and AFP level are also used in the survival assessment of HCC patients. However, it is generally believed that not only the characteristics of the tumor itself are closely connected with the prognosis of patients, but also host-related factors are closely related to the prognosis of the tumor. Thus, the above indicators lack a certain degree of sensitivity and specificity, nor do they consider the patient's nutritional status, inflammation, and other related factors.

Previous studies have shown that inflammation can promote the malignant biological behavior of liver cancer cells and is related to the prognosis of patients [11]. In recent years, several commonly used clinical indicators of inflammation have shown their potential as predictors of prognosis in various kinds of tumors. Neutrophil to lymphocyte ratio (NLR) is an indicator of systemic inflammation of humans, which might be related to the prognosis of many tumor patients including liver cancer [12]. Previous studies displayed that NLR is associated with prognosis of liver cancer patients undergoing radical resection [13] and radiofrequency ablation [14]. Besides, an increasing number of studies evaluated the value of platelet to lymphocyte ratio (PLR) and monocyte to lymphocyte ratio (MLR) in HCC and other tumors [15–17]. Therefore, it is reasonable to predict the prognosis of HCC patients using different inflammation indicators.

Albumin (ALB) and gamma-glutamyltransferase (GGT) are indicators of liver function and inflammation, respectively. New evidence suggested that serum ALB was an independent prognostic factor for several malignancies [18]. GGT is an essential enzyme that plays a role in the metabolism of glutathione. Numerous studies have shown that elevated GGT level was correlated with high carcinogenesis risk and poor outcome [19]. In fact, a researcher first proposed the concept of albumin to gamma-glutamyltransferase ratio (AGR) in 2017 and showed that AGR is a predictor of the prognosis of patients with intrahepatic cholangiocarcinoma [20]. Based on previous research, we thus reasonably hypothesized that the AGR might be a potential prognostic indicator for HCC patients receiving RFA.

Till now, no studies have proven the prognostic value of AGR in HCC patients undergoing RFA. Therefore, we sought to evaluate the significance of AGR in HCC and constructed an AGR-based nomogram to predict prognosis for HCC patients undergoing RFA as initial treatment.

## 2. Materials and Methods

### 2.1. Patients

From 2010 to 2018, 394 early-stage HCC patients who received RFA as initial treatment were retrospectively recruited in Zhongshan Hospital of Fudan University. The inclusion criteria of patients were as follows: (1) diagnosed with HCC according to the American Association for the Study of Liver Diseases (AASLD) criteria [21]; (2) CP grade A or B; (3) solitary nodule < 5 cm or ≤3 nodule and none >3 cm; (4) no evidence of vascular invasion, lymph node involvement, or extrahepatic metastasis; (5) there were preoperative laboratory data and complete follow-up data.

The exclusion criteria were as follows: (1) patients undergoing other antitumor treatments before RFA; (2) patients undergoing incomplete ablation confirmed by contrast-enhanced computed tomography (CT) or magnetic resonance imaging (MRI) scan 4 weeks after RFA; (3) patients with other malignant tumors at the same time; and (4) patients with chronic kidney disease stage ≥ III and heart failure with New York Heart Association class ≥ III. Subsequently, 394 HCC patients were randomly allocated into a training cohort (*n* = 279) and verification cohort (*n* = 115) at a ratio of 7 : 3. Patient clinical characteristics, including age, gender, etiology, presence or absence of liver cirrhosis, laboratory results, and tumor-related characteristics before initial RFA treatment, were collected. The AGR was calculated by the ratio of serum ALB (g/L) to GGT (U/L).

The present study got ethics approval from the Medical Ethics Committee of Zhongshan Hospital of Fudan University and complies with the Declaration of Helsinki.

### 2.2. Treatment and Follow-Up

The surgical procedure has been described in a previous study [22]. All patients were followed up one month after the initial RFA and every two or three months thereafter. A physical examination, blood routine examination, liver function examination, tumor maker examination, and abdominal ultrasound examination were performed. Contrast-enhanced CT or MRI was immediately performed on patients whose test results indicate tumor recurrence.

### 2.3. Statistical Analysis

Continuous variables were expressed in mean ± standard deviation (SD), and the Student *t*-test was used for comparison. Categorical variables were described by percentages and compared using Pearson *χ*^2^ analysis or Fisher's exact test. X-tile statistical software (version 3.6.1, Yale University, New Haven, CT, USA) was applied to determine the optimal threshold of AGR for OS. According to the defined cutoff value, patients were divided into a low-AGR group and high-AGR group. The survival curve was estimated by the Kaplan-Meier method, and the survival difference was estimated by the log-rank test. We also used univariate analysis to identify significant variables related to OS. We selected variables with *P* value less than 0.1 in multivariate Cox proportional hazards regression model. Concordance index (*C*-index), area under the curve (AUC), and likelihood ratio test (LAT) were used to compare the prediction effects of AGR, CP classification, and albumin-bilirubin (ALBI) classification. Combined with the results of multivariate analysis, an AGR-based nomograph of 3-year and 5-year OS proportion was constructed. The Hosmer-Lemeshow test (H-L test) and calibration curve were used to evaluate the degree of consistency between the predicted risk of the model and the actual risk. Stata software (version 15.1, StataCorp, College Station, TX) and R software (version 3.5.1) were utilized for analysis. *P* < 0.05 was thought as statistically significant.

## 3. Results

### 3.1. Demographics and Clinical Characteristics

Table 1 displays the main characteristics of the training cohort and validation cohort. In the training cohort, there were 219 males (78.5%) and 60 females (21.5%), with an average age of 58.58 ± 11.27 years. 224 patients (80.3%) had hepatitis B virus (HBV) infection background. There were 182 cases (65.2%) with liver cirrhosis and 277 cases (99.3%) with CP A grade. The laboratory results showed that the average levels of ALB and *γ*-GT were 39.43 ± 5.00 g/L and 74.64 ± 95.98 U/L, respectively. In terms of tumor characteristics, 47 cases (16.8%) had tumors larger than 3 cm in diameter, and 59 cases (21.1%) had multiple tumors. In the American Joint Committee on Cancer TNM staging system (AJCC-TNM) Ia, Ib, and II, there were 133 cases (47.7%), 87 cases (31.2%), 59 cases (21.1%), respectively.

In the validation cohort, there were 87 males (75.7%) and 28 females (24.3%), with an average age of 59.02 ± 11.52 years. 87 patients (75.7%) had hepatitis B virus (HBV) infection background. There were 77 cases (70.0%) with liver cirrhosis and 114 cases (99.1%) with CP A grade. The laboratory results showed that the average levels of ALB and *γ*-GT were 39.61 ± 8.59 g/L and 80.08 ± 94.03 U/L, respectively. In terms of tumor characteristics, 25 cases (21.7%) had tumors larger than 3 cm in diameter, and 20 cases (17.4%) had multiple tumors. In the AJCC-TNM Ia, Ib, and II, there were 47 cases (40.9%), 48 cases (41.7%), and 20 cases (17.4%), respectively. In a word, no statistical difference was found between the training cohort and validation cohort regarding demographics and clinical characteristics.

### 3.2. Clinicopathological Characteristics of the Low-AGR Group and High-AGR Group

By analyzing the data of the training cohort using X-tile, the optimal threshold of AGR was determined to be 0.63. Then, the patients were divided into the low-AGR group (AGR ≤ 0.63, *n* = 111) and high-AGR group (AGR > 0.63, *n* = 168). The correlation between AGR and other characteristics is shown in Table 2. Generally, patients with low AGR levels have poorer liver function (ALBI stages 2 and 3) and have higher levels of AKP (*P* < 0.05).

### 3.3. Survival Analysis

The mean follow-up time was 39.1 months (range: 1-140 months). At the end of the follow-up, 68 patients (17.3%) died and 296 patients (75.1%) had tumor recurrence. In the training cohort, the 3-year and 5-year overall survival (OS) rates were 84.9% and 72.6%, respectively. In the validation cohort, the 3-year, and 5-year OS rates were 83.3% and 62.9%, respectively. Regarding RFS, the 3-year and 5-year recurrence-free survival (RFS) rates of patients in the training cohort were 31.4% and 17.4%, respectively, while the 3-year and 5-year RFS rates were 30.7% and 13.0% in the validation cohort. What is more, there was no statistical difference in survival time between the two groups (*P* = 0.310 for OS and *P* = 0.356 for RFS).

**Figure 1:**
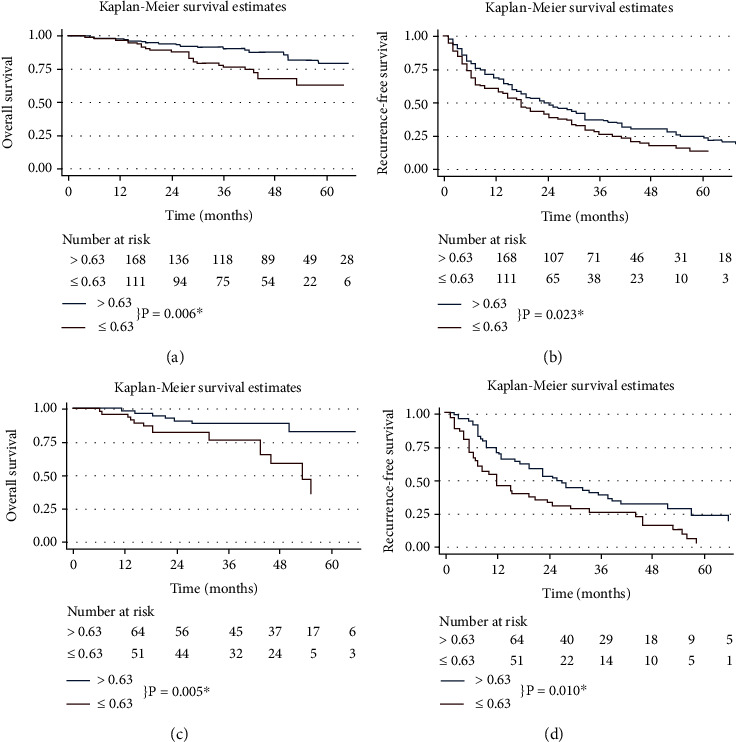
Kaplan–Meier survival curves of different groups divided by AGR. OS according to AGR values in the training cohort and validation cohort (a, c). RFS according to AGR values in the training cohort and validation cohort (b, d) ^∗^Statistically significant. Abbreviations: AGR: albumin to gamma-glutamyltransferase ratio; OS: overall survival; RFS: recurrence-free survival.

As shown in Figure 1, the survival rate of patients with low AGR levels was significantly lower than that of patients with high AGR levels. The 3-year and 5-year OS rates were 90.6% and 79.4% in the high-AGR group, and 76.7% and 63.3% in the high-AGR group, respectively. The 3-year and 5-year RFS rates were 35.0% and 20.5% in the high-AGR group and 26.1% and 13.5% in the high-AGR group, respectively. In addition, the above findings have been verified in the verification cohort.

### 3.4. Univariate and Multivariate Analysis

In the training cohort, the outputs of univariate analysis showed that AGR, AKP, and tumor size were significantly associated with OS. Through further multivariate analysis, the above factors were all important independent factors affecting prognosis. In the validation cohort, univariate and multivariate analyses confirmed that AGR, AKP, and tumor size are index of OS in HCC patients. The details are illustrated in Table 3.

### 3.5. Comparing the Prognostic Performance of AGR with Different Liver Function Assessment Methods

**Figure 2:**
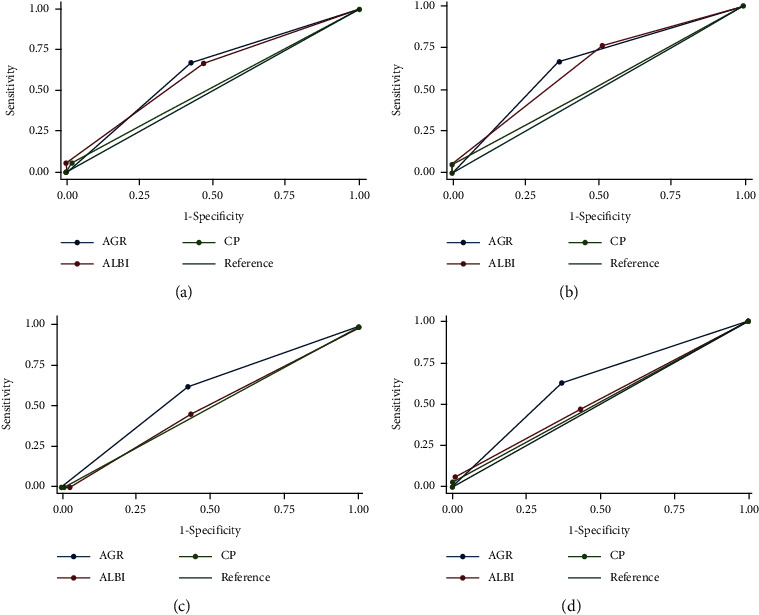
ROC curves of AGR, ALBI grade, and CP grade: (a, b) ROC curves for predicting the 3-year and 5-year OS in the training cohort; (c, d) ROC curves for predicting the 3-year and 5-year OS in the validation cohort. Abbreviations: ROC: receiver operating characteristic; AGR: albumin to gamma-glutamyltransferase ratio; ALBI: albumin-bilirubin; CP: Child-Pugh; OS: overall survival.

The prognostic prediction effects of AGR, AGR grade, and CP classification were evaluated by *C*-index, AUCs, and LAT *χ*^2^ values. In the training cohort, the *C*-index values of AGR, CP classification, and ALBI grade were 0.64 (95% CI: 0.49-0.78), 0.61 (95% CI: 0.48-0.76), and 0.52 (95% CI: 0.37-0.68), while in the validation cohort, the corresponding *C*-index values were 0.62 (95% CI: 0.50-0.74), 0.52 (95% CI: 0.42-0.64), and 0.50 (95% CI: 0.38-0.62), respectively. We have also noticed that the 3-year and 5-year AUCs and LAT *χ*^2^ of AGR were higher than those of ALBI and CP in both the training cohort and validation cohort. All these results indicated that AGR can be used as a superior predictor for prognosis in HCC patients undergoing RFA. More details are demonstrated in Table 4 and Figure 2.

### 3.6. Development and Validation of AGR-Based Nomogram Model

**Figure 3:**
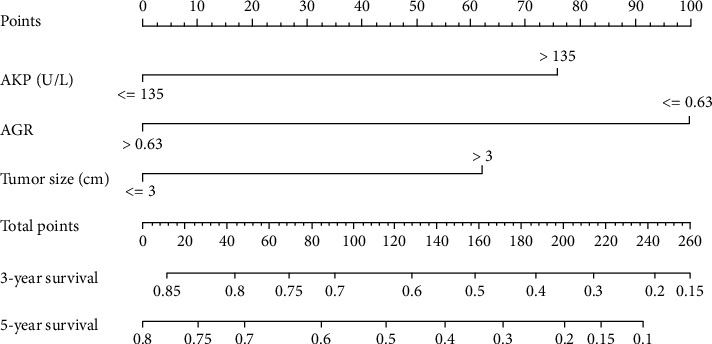
The AGR-based nomogram for forecasting the 3-year and 5-year survival probabilities of HCC after RFA. Abbreviations: AGR: albumin to gamma-glutamyltransferase ratio; HCC: hepatocellular carcinoma; RFA: radiofrequency ablation.

Through the multivariate Cox proportional hazards model, three variables including AGR, AKP, and tumor size were determined as independent prognostic factors. Based on those factors, we constructed the 3- and 5-year OS forecast nomogram model, shown in Figure 3.

**Figure 4:**
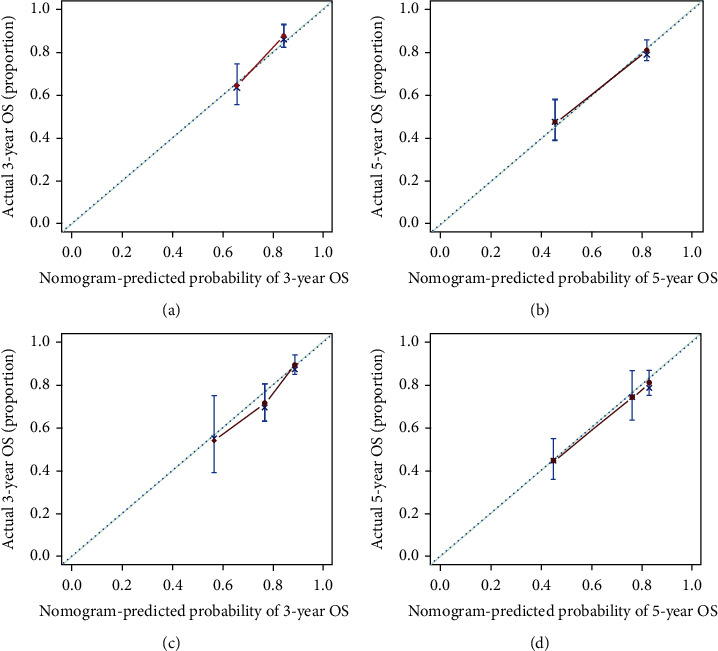
Calibration curves for predicting the 3-year and 5-year OS of HCC patients receiving RFA. Calibration curves for predicting the 3-year and 5-year OS in the training cohort (a, b) and validation cohort (c, d). Abbreviations: HCC: hepatocellular carcinoma; RFA: radiofrequency ablation; OS: overall survival.

In addition, we performed the H-L test and calibration curves to verify the value of the AGR-based nomogram. The *P* values of the H-L test for 3-year and 5-year OS in the training cohort were 0.837 and 0.963, respectively. In the validation cohort, the corresponding *P* values were 0.630 and 0.942. All *P* values were above 0.05, indicating a good fit of the AGR-based nomogram. Besides, the calibration curves showed that the predicted probabilities of 3-year and 5-year OS of the AGR-based nomogram were close to the actual probabilities, suggesting AGR-based nomogram had an excellent predictive value in the training or validation cohort (Figure 4).

## 4. Discussion

HCC is one of the common malignant tumors of the digestive system that seriously endanger human health. The incidence and mortality of primary liver cancer in Asian population are higher than those in other populations [23]. RFA is an effective method for the management of small liver cancer. Evaluating the survival time of HCC patients after treatment is of great significance for formulating reasonable treatment plans and improving the quality of life of patients with HCC.

In the present study, we first confirmed the prognostic value of AGR in HCC patients receiving RFA as the initial treatment. The AGR of 0.63 was determined as the best cutoff value in the present study. Patients with low levels of AGR are significantly detrimental to OS and RFS in the training and validation group. In addition, our results comprehensively indicate that preoperative AGR is an important prognostic indicator of HCC undergoing RFA.

Chronic inflammation is one of the main factors that promote the occurrence or development of tumors. It is believed about twenty percent of malignant tumors develop from inflammation [24]. Once inflammation is triggered, inflammatory cells will secrete a large number of inflammatory factors in the process of migrating, causing damage to DNA and destroying the stability of proliferating cells' genes. Finally, under the repeated stimulation of inflammatory factors, the genes of the cells change and unrestricted proliferation occurs. And the upregulated cytokine can further promote angiogenesis and tumor metastasis [25]. After liver cells are damaged, the cell membrane ruptures or increases in permeability, causing liver enzymes to escape into the blood. Peripheral blood liver enzymes, such as GGT, are the final manifestation of liver inflammation in the peripheral blood, which can better reflect the symptoms of hepatitis. A comprehensive analysis of inflammation-related cells or liver enzyme indicators in peripheral blood should be able to reflect the liver inflammation more objectively and accurately, so as to judge the prognosis of liver cancer.

There is no doubt that liver function is a key factor in the prognosis of systemic diseases. In detail, ALB is a kind of protein synthesized by the liver cells, reflecting the nutritional status of patients. When the patient's body is stimulated by tumor cells and inflammatory factors, the ability of the liver to synthesize albumin is significantly reduced, and the content of serum albumin will be significantly reduced. Thus, as an important liver function index, ALB is one of the indexes of CP classification system. Studies have shown that ALB was related to the prognosis of various malignant tumors, such as gastric cancer, colon cancer, liver cancer, and glioblastoma [26, 27].

Based on the above, AGR is not just a combination of liver function parameters; it is more a reflection of the internal inflammation state and seems to help assess the survival of cancer patients. In fact, Jing et al. [20] analyzed the AGR levels of 206 patients with intrahepatic cholangiocarcinoma for the first time. They calculated that the optimal cutoff value of AGR for patients with cholangiocarcinoma was 0.6. Recently, AGR was also used to forecast the prognosis of gallbladder cancer [28]. By analyzing the preoperative AGR levels of 140 patients with gallbladder cancer, researchers established and verified the prediction nomograms of 1-, 3-, and 5-year survival probability. In terms of consistency, identification, and net benefits, AGR-based nomograms achieved considerable prognostic performance. AGR also showed a good predictive value in pancreatic ductal adenocarcinoma [29]. In the current study, we firstly verified that AGR might be an indicator for forecasting the prognosis of HCC patients with RFA as initial treatment.

As far as we know, CP classification and ALBI grade are common tools for the assessment of liver function and patients' prognosis [30, 31]. However, CP classification was originally established on the basis of predicting the prognosis of esophageal gastric varix devascularization in patients with cirrhosis and portal hypertension [32]. CP classification includes serum albumin, bilirubin, and prothrombin time but also includes the presence of hepatic encephalopathy and ascites, so the results obtained by CP classification contain a certain degree of subjectivity. Therefore, the application of CP classification to assess liver function in patients with HCC has always been controversial. ALBI grade is developed on the basis of a large number of cohort studies of HCC patients and is a more objective method for evaluating liver function [33]. Unlike other malignant tumors, most primary liver cancer in China is caused by chronic liver disease and hepatitis B virus infection. What is more, a previous study found that the level of serum GGT had a potential to predict the outcome of primary liver cancer patients after RFA [34]. Therefore, we selected AGR, an indicator of liver function and inflammation, to try to predict the prognosis of HCC patients receiving RFA as the initial treatment. Unfortunately, based on multivariate analysis, neither CP grade nor ALBI grade in HCC showed good prognostic significance. In contrast, AGR was determined as an independent prognostic factor for both the training group and validation cohort. In addition, the *C*-index, AUC, and LAT *χ*^2^ values proved that AGR was more discriminatory compared with the CP grade and ALBI grade in HCC. Based on the results of univariate and multivariate analyses, we further established an AGR-based prognostic nomogram, which might be helpful for clinical decision making. Moreover, we further evaluated the predictive performance of the nomogram. The results of the calibration curves suggested that the nomogram had good predictive performance in both the training cohort and validation cohort.

However, there are some limitations in the current study. Firstly, the current study is a retrospective research. Besides, the sample size is relatively limited. And there are many factors that affect AGR. Therefore, strict control of bias and more detailed stratification are needed. Moreover, more patients and multicenter study are needed to verify our conclusions and obtain more reliable results.

To sum up, we found that HCC patients undergoing RFA as initial treatment with AGR ≥ 0.63 have a better prognosis than those with AGR < 0.63. The AGR-based nomogram model can effectively predict prognosis of early HCC patients undergoing RFA.

## Figures and Tables

**Table 1:** Demographics and clinical characteristics of patients in the training and validation cohorts.

	Total (*n* = 394)	Training cohort (*n* = 279)	Validation cohort (*n* = 115)	*P* value^∗^
Gender				0.538
Male	306	219	87	
Female	88	60	28	
Age	58.71 ± 11.33	58.58 ± 11.27	59.02 ± 11.52	0.726
TB (*μ*mol/L)	14.23 ± 7.81	13.88 ± 7.83	15.05 ± 7.75	0.173
ALT (U/L)	46.96 ± 61.28	43.01 ± 46.45	56.50 ± 86.82	0.117
PT	12.89 ± 2.17	12.85 ± 2.13	12.99 ± 2.28	0.564
ALB	39.48 ± 6.26	39.43 ± 5.00	39.61 ± 8.59	0.793
AFP	328.78 ± 1164.07	274.61 ± 816.44	460.20 ± 1738.18	0.275
*γ*-GT (U/L)	76.25 ± 95.40	74.64 ± 95.98	80.08 ± 94.03	0.363
Etiology				0.544
HBV infection	311	224	87	
HCV infection	13	8	5	
Other	70	47	23	
Liver cirrhosis				0.743
Yes	259	182	77	
No	135	97	38	
AKP (U/L)	94.54 ± 52.08	92.18 ± 44.41	100.27 ± 67.06	0.236
AGR	0.99 ± 0.74	1.00 ± 0.73	0.98 ± 0.77	0.806
CP grade				0.874
A	394	277	114	
B	3	2	1	
AGR				0.403
1	232	168	64	
2	162	111	51	
*γ*-GT (U/L)				0.719
≤50	195	140	55	
>50	199	139	60	
Tumor size (cm)				0.253
≤3	322	232	90	
3-5	72	47	25	
Tumor number				0.397
Single	315	220	95	
Multiple	79	59	20	
AJCC TNM-8				0.137
Ia	180	133	47	
Ib	135	87	48	
II	79	59	20	
ALBI				0.264
1	225	166	59	
2	164	109	55	
3	5	4	1	

Abbreviations: AGR: albumin to gamma-glutamyltransferase ratio; TB: total bilirubin; ALT: alanine aminotransferase; PT: prothrombin time; ALB: albumin; AFP: alfa-fetoprotein; *γ*-GT: *γ*-glutamyl transpeptidase; HBV: hepatitis B virus; HCV: hepatitis C virus; AKP: alkaline phosphatase; CP grade: Child-Pugh grade; AJCC TNM-8: the 8th edition of American Joint Committee on Cancer TNM staging system; ALBI grade: albumin-bilirubin grade. ^∗^Statistically significant.

**Table 2:** Associations between AGR and other characteristics.

	Total (*n* = 279)	AGR > 0.63 (*n* = 168)	AGR ≤ 0.63 (*n* = 111)	*P* value^∗^
Gender				0.737
Male	219	133	86	
Female	60	35	25	
Age	58.58 ± 11.27	57.76 ± 11.42	59.82 ± 10.97	0.136
TB (*μ*mol/L)	13.88 ± 7.83	13.25 ± 8.19	14.84 ± 7.17	0.098
ALT (U/L)	43.01 ± 46.45	40.46 ± 45.34	46.98 ± 45.94	0.301
PT	12.85 ± 2.13	12.79 ± 1.75	12.96 ± 2.60	0.501
ALB	39.43 ± 5.00	40.68 ± 4.52	37.52 ± 5.11	<0.001^∗^
AFP	274.61 ± 816.44	316.25 ± 882.14	211.58 ± 704.68	0.295
*γ*-GT (U/L)	73.44 ± 95.98	34.48 ± 13.69	132.41 ± 131.04	<0.001^∗^
Etiology				0.272
HBV infection	224	139	86	
HCV infection	8	6	2	
Other	47	24	23	
Liver cirrhosis				0.916
Yes	182	110	72	
No	97	58	39	
AKP (U/L)	92.18 ± 44.41	80.42 ± 27.57	109.99 ± 57.44	<0.001^∗^
AGR	1.00 ± 0.73	1.41 ± 0.67	0.38 ± 0.15	<0.001^∗^
CP grade				0.081
A	277	168	109	
B	2	0	2	
*γ*-GT (U/L)				<0.001^∗^
≤50	140	139	1	
>50	139	29	110	
Tumor size (cm)				0.281
≤3	232	143	89	
3-5	47	25	22	
Tumor number				0.175
Single	220	137	83	
Multiple	59	31	28	
AJCC TNM-8				0.200
Ia	133	87	46	
Ib	87	50	37	
II	59	31	28	
ALBI				<0.001^∗^
1	166	115	51	
2	109	53	56	
3	4	0	4	

Abbreviations: AGR: albumin to gamma-glutamyltransferase ratio; TB: total bilirubin; ALT: alanine aminotransferase; PT: prothrombin time; ALB: albumin; AFP: alfa-fetoprotein; *γ*-GT: *γ*-glutamyl transpeptidase; HBV: hepatitis B virus; HCV: hepatitis C virus; AKP: alkaline phosphatase; CP grade: Child-Pugh grade; AJCC TNM-8: the 8th edition of American Joint Committee on Cancer TNM staging system; ALBI grade: albumin-bilirubin grade. ^∗^Statistically significant.

**Table 3:** Univariate and multivariate analyses for overall survival in the training cohort and validation cohort.

Variable	Training cohort			Validation cohort		
	Univariate analysis	Multivariate analysis	*P* value	Univariate analysis	Multivariate analysis	*P* value
	*P* value	HR (95% CI)		*P* value	HR (95% CI)	
Age (>65)	0.298			0.781		
Gender (female/male)	0.370			0.451		
HBV infection (presence)	0.875			0.389		
HCV infection (presence)	0.274			0.253		
Liver cirrhosis (no/yes)	0.234			0.631		
AGR stage	0.012^∗^	2.295 (1.248-4.221)	0.008^∗^	0.024^∗^	3.327 (1.349-8.206)	0.009^∗^
AKP (U/L) (>135)	0.031^∗^	3.299 (1.458-7.467)	0.004^∗^	0.039^∗^	3.409 (1.317-8.825)	0.012^∗^
CP grade (A vs. B)	0.024^∗^			0.245		
Tumor number (single, multiple)	0.485			0.466		
Tumor size (cm) (≤3, 3–5)	0.044^∗^	2.002 (1.108-3.974)	0.047^∗^	0.021^∗^	2.090 (1.223-3.573)	0.007^∗^
TB (*μ*mol/L) (≥34)	0.952			0.441		
ALT (U/L) (>40)	0.364			0.682		
AFP (ng/mL) (>400)	0.624			0.570		
ALBI grade (1/2/3)	0.017^∗^			0.111		
AJCC TNM-8 (Ia, Ib, II)	0.945			0.307		

Abbreviations: HR: hazard ratio; HBV: hepatitis B virus; HCV: hepatitis C virus; AGR: albumin to gamma-glutamyltransferase ratio; AKP: alkaline phosphatase; CP grade: Child-Pugh grade; TB: total bilirubin; ALT: alanine aminotransferase; AFP: alfa-fetoprotein; ALBI grade: albumin-bilirubin grade; AJCC TNM-8: the 8th edition of American Joint Committee on Cancer TNM staging system. ^∗^Statistically significant.

**Table 4:** Comparison of predictive efficacy among different liver function-related indices.

	Training cohort				Validation cohort			
	*C*-index	AUC (95% CI)		LAT *χ*^2^	*C*-index	AUC (95% CI)		LAT *χ*^2^
		3-year OS	5-year OS			3-year OS	5-year OS	
AGR	0.64 (0.49-0.78)	0.63 (0.48-0.76)	0.65 (0.52-0.78)	11.2	0.62 (0.50-0.74)	0.59 (0.50-0.69)	0.63 (0.52-0.73)	6.7
ALBI grade	0.61 (0.48-0.76)	0.61 (0.46-0.76)	0.63 (0.50-0.77)	4.9	0.52 (0.42-0.64)	0.51 (0.41-0.61)	0.53 (0.43-0.64)	3.1
CP grade	0.52 (0.37-0.68)	0.52 (0.36-0.67)	0.53 (0.38-0.68)	2.2	0.50 (0.38-0.62)	0.49 (0.38-0.57)	0.52 (0.40-0.63)	0.7

Abbreviations: AUC: area under the curve; LAT *χ*^2^; likelihood ratio test *χ*^2^; OS: overall survival; AGR: albumin to gamma-glutamyltransferase ratio; ALBI grade: albumin-bilirubin grade; CP grade: Child-Pugh grade. ^∗^Statistically significant.

## Data Availability

All data is available on request.
